# Dimethyl (2-hydr­oxy-4-phenyl­but-3-en-2-yl)phospho­nate

**DOI:** 10.1107/S1600536809044067

**Published:** 2009-10-31

**Authors:** M. Nawaz Tahir, Nurcan Acar, Hamza Yilmaz, Riaz H. Tariq

**Affiliations:** a Department of Physics, University of Sargodha, Sargodha, Pakistan; b Department of Chemistry, Faculty of Science, University of Ankara, Ankara, Turkey; c Institute of Chemical and Pharmaceutical Sciences, The University of Faisalabad, Faisalabad, Pakistan

## Abstract

In the title compound, C_12_H_17_O_4_P, the phenyl­butenyl group is disordered over two sets of sites with an occupancy ratio of 0.755 (12):0.245 (12). In the crystal, inversion dimers linked by pairs of O—H⋯O hydrogen bonds occur, forming *R*_2_^2^(10) ring motifs. The packing is consolidated by weak C—H⋯π inter­actions.

## Related literature

For related structures, see: Acar *et al.* (2009 ▶); Tahir *et al.* (2007 ▶, 2009*a* ▶,*b* ▶). For graph-set theory, see: Bernstein *et al.* (1995 ▶).
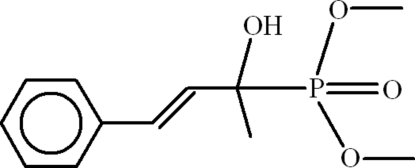


## Experimental

### 

#### Crystal data

C_12_H_17_O_4_P
*M* *_r_* = 256.23
Monoclinic, 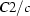

*a* = 17.1522 (12) Å
*b* = 8.1571 (13) Å
*c* = 19.5230 (12) Å
β = 103.771 (10)°
*V* = 2653.0 (5) Å^3^
*Z* = 8
Mo *K*α radiation
μ = 0.21 mm^−1^
*T* = 296 K
0.25 × 0.14 × 0.12 mm

#### Data collection

Enraf–Nonius CAD-4 diffractometer
Absorption correction: ψ scan (*MolEN*; Fair, 1990 ▶) *T*_min_ = 0.885, *T*_max_ = 0.954
2519 measured reflections
2415 independent reflections
1684 reflections with *I* > 2σ(*I*)
*R*_int_ = 0.095
3 standard reflections frequency: 120 min intensity decay: 0.6%

#### Refinement

*R*[*F* ^2^ > 2σ(*F* ^2^)] = 0.061
*wR*(*F* ^2^) = 0.175
*S* = 1.07
2415 reflections
177 parameters
H-atom parameters constrained
Δρ_max_ = 0.53 e Å^−3^
Δρ_min_ = −0.42 e Å^−3^

### 

Data collection: *CAD-4 EXPRESS* (Enraf–Nonius, 1993 ▶); cell refinement: *CAD-4 EXPRESS*; data reduction: *MolEN* (Fair, 1990 ▶); program(s) used to solve structure: *WinGX* (Farrugia, 1999 ▶); program(s) used to refine structure: *SHELXL97* (Sheldrick, 2008 ▶); molecular graphics: *ORTEP-3 for Windows* (Farrugia, 1997 ▶) and *PLATON* (Spek, 2009 ▶); software used to prepare material for publication: *WinGX* (Farrugia, 1999 ▶).

## Supplementary Material

Crystal structure: contains datablocks global, I. DOI: 10.1107/S1600536809044067/hb5172sup1.cif

Structure factors: contains datablocks I. DOI: 10.1107/S1600536809044067/hb5172Isup2.hkl

Additional supplementary materials:  crystallographic information; 3D view; checkCIF report

## Figures and Tables

**Table 1:** Hydrogen-bond geometry (Å, °)

*D*—H⋯*A*	*D*—H	H⋯*A*	*D*⋯*A*	*D*—H⋯*A*
O1—H1⋯O2^i^	0.82	1.90	2.721 (3)	176
C6*B*—H6*B*⋯*Cg*1^ii^	0.93	2.83	3.568 (17)	137
C6*B*—H6*B*⋯*Cg*2^ii^	0.93	2.94	3.652 (17)	134

Symmetry codes: (i) 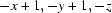; (ii) 
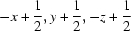
. *Cg*1 and *Cg*2 are the centroids of the C1*A*–C6*A* and C1*B*–C6*B* rings, respectively.

**Fig. 1.**
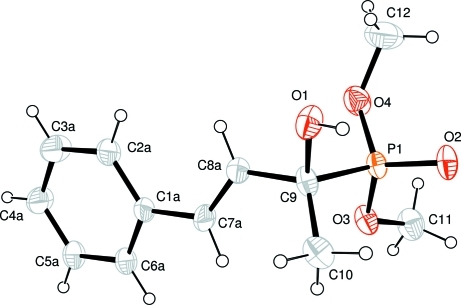
View of (I) with the atom numbering scheme for atoms of greater occupancy ratio. The displacement ellipsoids are drawn at the 30% probability level. H-atoms are shown by small circles of arbitrary radii.

**Fig. 2.**
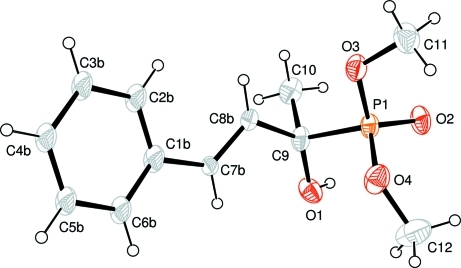
View of the title compound with the atom numbering scheme for atoms of smaller occupancy ratio. The displacement ellipsoids are drawn at the 30% probability level. H-atoms are shown by small circles of arbitrary radii.

**Fig. 3.**
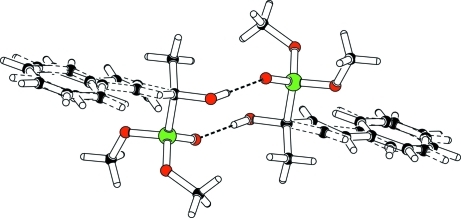
The partial packing of (I), which shows that molecules form inversion dimers.

## supplementary crystallographic information

### Comment

We have reported the crystal structures which contains α-hydroxy phosphonate such as (II) Dimethyl (1-hydroxy-1,2-diphenylethyl)phosphonate (Tahir *et al.*, 2009*a*), (III) Dimethyl [hydroxy(2-nitrophenyl)methyl]phosphonate (Tahir *et al.*, 2009*b*), (IV) (*R*)-Dimethyl [(2-chlorophenyl)hydroxymethyl]phosphonate (Tahir *et al.*, 2007) and Diethyl (1-hydroxy-1,2-diphenylethyl)phosphonate (Acar *et al.*, 2009). The title compound (I, Fig. 1) is in continuation of synthesizing various α-hydroxy phosphonates.

In the crystal structure of title compound phenylbutan is disordered over two possible sites with occupancy ratio of 0.755 (12):0.245 (12). The benzene rings of disordered moieties A (C1A—C6A) and B (C1B—C6B) are nearly planar to one another as the dihedral angle between A/B is 2.76 (1.35)°. The molecules of title compound are dimerized due to O—H···O type of intermolecular H-bondings (Table 1, Fig. 3) forming *R*_2_^2^(10) ring motif (Bernstein *et al.*, 1995). The molecules are stabilized due to C–H···π interactions (Table 1).

### Experimental

Benzylacetone (4.45 g, 30 mmol) and dimethylphosphonate (3.30 g, 30 mmol) were dissolved in 50 ml of tetrahydrofuran. The mixture was cooled to 273 K and in it KF (1.74 g, 30 mmol) and γ-Al_2_O_3_ (1.74 g, 17 mmol) were added and refluxed. The precipitates obtained after 48 h were washed with hot distiled water (50 ml) and dried. The crude material was dissolved in distiled water with few drops of ethyl alcohol and colourless needles of (I) were obtained after 4 days.

### Refinement

The H-atoms were positioned geometrically (O–H = 0.82 Å, C–H = 0.93-0.96 Å) and refined as riding with *U*_iso_(H) = 1.2*U*_eq_(carrier) or 1.5*U*_eq_(methyl C).

The incorrect bond distances and higher thermal parameters of phenylbutan lead to disorder. In the disordered group the benzene rings were refined using *AFIX 66*. The benzene ring B (C2B—C6B) was refined using EADP.

### Crystal data

**Table:**

C_12_H_17_O_4_P	*F*(000) = 1088
*M**_r_* = 256.23	*D*_x_ = 1.283 Mg m^−^^3^
Monoclinic, *C*2/*c*	Mo *K*α radiation, λ = 0.71073 Å
Hall symbol: -C 2yc	Cell parameters from 25 reflections
*a* = 17.1522 (12) Å	θ = 9.9–13.9°
*b* = 8.1571 (13) Å	µ = 0.21 mm^−^^1^
*c* = 19.5230 (12) Å	*T* = 296 K
β = 103.771 (10)°	Needle, colourless
*V* = 2653.0 (5) Å^3^	0.25 × 0.14 × 0.12 mm
*Z* = 8	

### Data collection

**Table:**

Enraf–Nonius CAD-4 diffractometer	*R*_int_ = 0.095
ω/2θ scans	θ_max_ = 25.5°, θ_min_ = 2.8°
Absorption correction: ψ scan (*MolEN*; Fair, 1990)	*h* = −20→20
*T*_min_ = 0.885, *T*_max_ = 0.954	*k* = 0→9
2519 measured reflections	*l* = −23→0
2415 independent reflections	3 standard reflections every 120 min
1684 reflections with *I* > 2σ(*I*)	intensity decay: 0.6%

### Refinement

**Table:**

Refinement on *F*^2^	Primary atom site location: structure-invariant direct methods
Least-squares matrix: full	Secondary atom site location: difference Fourier map
*R*[*F*^2^ > 2σ(*F*^2^)] = 0.061	H-atom parameters constrained
*wR*(*F*^2^) = 0.175	*w* = 1/[σ^2^(*F*_o_^2^) + (0.114*P*)^2^] where *P* = (*F*_o_^2^ + 2*F*_c_^2^)/3
*S* = 1.07	(Δ/σ)_max_ < 0.001
2415 reflections	Δρ_max_ = 0.53 e Å^−^^3^
177 parameters	Δρ_min_ = −0.42 e Å^−^^3^
0 restraints	

### Special details

**Table:**

Geometry. Bond distances, angles *etc*. have been calculated using the rounded fractional coordinates. All su's are estimated from the variances of the (full) variance-covariance matrix. The cell e.s.d.'s are taken into account in the estimation of distances, angles and torsion angles

### Fractional atomic coordinates and isotropic or equivalent isotropic displacement parameters (Å^2^)

**Table:**

	*x*	*y*	*z*	*U*_iso_*/*U*_eq_	Occ. (<1)
P1	0.43961 (4)	0.29251 (9)	0.06739 (4)	0.0399 (3)	
O1	0.43699 (13)	0.6068 (3)	0.08493 (11)	0.0548 (8)	
O2	0.49603 (12)	0.2946 (3)	0.02130 (12)	0.0528 (8)	
O3	0.37510 (12)	0.1526 (2)	0.05085 (11)	0.0500 (7)	
O4	0.48115 (13)	0.2636 (3)	0.14713 (12)	0.0597 (8)	
C1A	0.2124 (3)	0.4228 (6)	0.1674 (2)	0.0453 (16)	0.755 (12)
C2A	0.23963 (19)	0.4970 (7)	0.2329 (3)	0.058 (2)	0.755 (12)
C3A	0.1939 (3)	0.4884 (9)	0.28271 (18)	0.078 (2)	0.755 (12)
C4A	0.1209 (3)	0.4058 (9)	0.2671 (3)	0.072 (2)	0.755 (12)
C5A	0.0937 (2)	0.3316 (6)	0.2017 (3)	0.062 (2)	0.755 (12)
C6A	0.1395 (3)	0.3401 (6)	0.15183 (15)	0.0524 (17)	0.755 (12)
C7A	0.2601 (3)	0.4283 (6)	0.1139 (2)	0.0451 (16)	0.755 (12)
C8A	0.3344 (4)	0.4814 (7)	0.1215 (3)	0.0414 (19)	0.755 (12)
C9	0.37966 (18)	0.4799 (3)	0.06310 (16)	0.0440 (10)	
C10	0.3323 (2)	0.5090 (4)	−0.01138 (18)	0.0584 (11)	
C11	0.4001 (2)	−0.0152 (4)	0.0447 (2)	0.0644 (13)	
C12	0.5626 (2)	0.3125 (6)	0.1775 (2)	0.0896 (19)	
C6B	0.1066 (9)	0.341 (2)	0.1847 (9)	0.061 (3)	0.245 (12)
C7B	0.3065 (12)	0.441 (2)	0.0916 (13)	0.046 (6)	0.245 (12)
C1B	0.1670 (11)	0.380 (2)	0.1509 (5)	0.061 (3)	0.245 (12)
C2B	0.2328 (8)	0.471 (2)	0.1857 (9)	0.061 (3)	0.245 (12)
C3B	0.2381 (8)	0.525 (2)	0.2543 (9)	0.061 (3)	0.245 (12)
C4B	0.1777 (12)	0.486 (3)	0.2880 (7)	0.061 (3)	0.245 (12)
C5B	0.1119 (10)	0.395 (3)	0.2532 (9)	0.061 (3)	0.245 (12)
C8B	0.2981 (10)	0.5086 (19)	0.1470 (10)	0.057 (6)	0.245 (12)
H1	0.45706	0.63143	0.05218	0.0821*	
H2A	0.28844	0.55226	0.24331	0.0697*	0.755 (12)
H10C	0.36812	0.51292	−0.04233	0.0875*	
H11A	0.35594	−0.08762	0.04446	0.0966*	
H11B	0.41720	−0.02795	0.00165	0.0966*	
H11C	0.44381	−0.04117	0.08403	0.0966*	
H12A	0.59764	0.26187	0.15202	0.1344*	
H12B	0.56693	0.42950	0.17485	0.1344*	
H12C	0.57762	0.27862	0.22598	0.1344*	
H3A	0.21203	0.53806	0.32650	0.0928*	0.755 (12)
H4A	0.09026	0.40008	0.30047	0.0865*	0.755 (12)
H5A	0.04489	0.27630	0.19124	0.0741*	0.755 (12)
H6A	0.12130	0.29050	0.10804	0.0629*	0.755 (12)
H7A	0.23485	0.38956	0.06932	0.0541*	0.755 (12)
H8A	0.36086	0.52242	0.16532	0.0500*	0.755 (12)
H10A	0.29444	0.42147	−0.02555	0.0875*	
H10B	0.30398	0.61121	−0.01371	0.0875*	
H1B	0.16342	0.34369	0.10503	0.0729*	0.245 (12)
H3B	0.28213	0.58635	0.27758	0.0729*	0.245 (12)
H4B	0.18126	0.52218	0.33391	0.0729*	0.245 (12)
H5B	0.07147	0.36876	0.27580	0.0729*	0.245 (12)
H6B	0.06255	0.27951	0.16136	0.0729*	0.245 (12)
H7B	0.26815	0.36654	0.06805	0.0543*	0.245 (12)
H8B	0.33514	0.58922	0.16626	0.0680*	0.245 (12)

### Atomic displacement parameters (Å^2^)

**Table:**

	*U*^11^	*U*^22^	*U*^33^	*U*^12^	*U*^13^	*U*^23^
P1	0.0354 (4)	0.0436 (5)	0.0463 (5)	0.0019 (3)	0.0206 (3)	0.0024 (3)
O1	0.0676 (15)	0.0482 (12)	0.0568 (13)	−0.0064 (11)	0.0312 (12)	−0.0074 (10)
O2	0.0520 (13)	0.0542 (13)	0.0644 (14)	0.0030 (10)	0.0382 (11)	0.0013 (10)
O3	0.0399 (12)	0.0423 (11)	0.0723 (15)	0.0011 (9)	0.0225 (10)	−0.0003 (10)
O4	0.0499 (13)	0.0785 (15)	0.0530 (14)	0.0069 (12)	0.0167 (11)	0.0118 (11)
C1A	0.037 (3)	0.064 (3)	0.036 (2)	0.007 (2)	0.011 (2)	−0.005 (2)
C2A	0.043 (3)	0.093 (4)	0.045 (4)	−0.002 (2)	0.022 (2)	−0.011 (3)
C3A	0.055 (4)	0.140 (5)	0.047 (3)	−0.025 (4)	0.031 (3)	−0.022 (3)
C4A	0.053 (4)	0.121 (5)	0.051 (3)	−0.013 (3)	0.029 (3)	−0.020 (3)
C5A	0.046 (3)	0.096 (4)	0.054 (4)	0.000 (2)	0.033 (2)	−0.008 (3)
C6A	0.035 (3)	0.078 (3)	0.051 (3)	−0.002 (2)	0.024 (2)	−0.007 (2)
C7A	0.040 (3)	0.061 (3)	0.040 (2)	−0.007 (2)	0.021 (2)	−0.011 (2)
C8A	0.038 (4)	0.047 (3)	0.040 (3)	0.002 (2)	0.011 (3)	−0.005 (2)
C9	0.0388 (16)	0.0440 (16)	0.0560 (18)	0.0029 (13)	0.0245 (14)	0.0051 (13)
C10	0.051 (2)	0.0560 (19)	0.069 (2)	0.0017 (15)	0.0160 (17)	0.0091 (16)
C11	0.066 (2)	0.0462 (18)	0.085 (3)	0.0049 (16)	0.026 (2)	−0.0011 (16)
C12	0.065 (3)	0.119 (4)	0.074 (3)	−0.015 (2)	−0.005 (2)	−0.003 (2)
C6B	0.037 (4)	0.112 (7)	0.040 (5)	−0.002 (4)	0.024 (3)	−0.003 (4)
C7B	0.034 (10)	0.047 (9)	0.058 (13)	0.003 (7)	0.015 (9)	0.002 (7)
C1B	0.037 (4)	0.112 (7)	0.040 (5)	−0.002 (4)	0.024 (3)	−0.003 (4)
C2B	0.037 (4)	0.112 (7)	0.040 (5)	−0.002 (4)	0.024 (3)	−0.003 (4)
C3B	0.037 (4)	0.112 (7)	0.040 (5)	−0.002 (4)	0.024 (3)	−0.003 (4)
C4B	0.037 (4)	0.112 (7)	0.040 (5)	−0.002 (4)	0.024 (3)	−0.003 (4)
C5B	0.037 (4)	0.112 (7)	0.040 (5)	−0.002 (4)	0.024 (3)	−0.003 (4)
C8B	0.036 (8)	0.056 (9)	0.083 (12)	−0.010 (7)	0.023 (9)	−0.028 (8)

### Geometric parameters (Å, °)

**Table:**

P1—O2	1.470 (2)	C8A—C9	1.525 (7)
P1—O3	1.569 (2)	C9—C10	1.506 (5)
P1—O4	1.568 (2)	C1B—H1B	0.9300
P1—C9	1.833 (3)	C2A—H2A	0.9300
O1—C9	1.422 (4)	C3A—H3A	0.9300
O3—C11	1.448 (4)	C3B—H3B	0.9300
O4—C12	1.438 (4)	C4A—H4A	0.9300
O1—H1	0.8200	C4B—H4B	0.9300
C1A—C2A	1.391 (7)	C5A—H5A	0.9300
C1A—C7A	1.473 (7)	C5B—H5B	0.9300
C1A—C6A	1.389 (7)	C6A—H6A	0.9300
C1B—C2B	1.39 (2)	C6B—H6B	0.9300
C1B—C6B	1.39 (2)	C7A—H7A	0.9300
C2A—C3A	1.389 (6)	C7B—H7B	0.9300
C2B—C8B	1.52 (2)	C8A—H8A	0.9300
C2B—C3B	1.39 (2)	C8B—H8B	0.9300
C3A—C4A	1.390 (8)	C10—H10B	0.9600
C3B—C4B	1.39 (2)	C10—H10A	0.9600
C4A—C5A	1.390 (8)	C10—H10C	0.9600
C4B—C5B	1.39 (3)	C11—H11A	0.9600
C5A—C6A	1.391 (6)	C11—H11B	0.9600
C5B—C6B	1.39 (2)	C11—H11C	0.9600
C7A—C8A	1.321 (9)	C12—H12A	0.9600
C7B—C8B	1.25 (3)	C12—H12B	0.9600
C7B—C9	1.52 (2)	C12—H12C	0.9600
			
O2—P1—O3	114.66 (13)	C2A—C3A—H3A	120.00
O2—P1—O4	113.56 (13)	C4A—C3A—H3A	120.00
O2—P1—C9	113.97 (14)	C2B—C3B—H3B	120.00
O3—P1—O4	103.09 (12)	C4B—C3B—H3B	120.00
O3—P1—C9	103.70 (12)	C3A—C4A—H4A	120.00
O4—P1—C9	106.75 (14)	C5A—C4A—H4A	120.00
P1—O3—C11	119.8 (2)	C3B—C4B—H4B	120.00
P1—O4—C12	122.3 (2)	C5B—C4B—H4B	120.00
C9—O1—H1	109.00	C4A—C5A—H5A	120.00
C2A—C1A—C7A	121.1 (4)	C6A—C5A—H5A	120.00
C6A—C1A—C7A	118.9 (4)	C4B—C5B—H5B	120.00
C2A—C1A—C6A	120.0 (4)	C6B—C5B—H5B	120.00
C2B—C1B—C6B	120.1 (12)	C5A—C6A—H6A	120.00
C1A—C2A—C3A	120.0 (4)	C1A—C6A—H6A	120.00
C1B—C2B—C3B	120.0 (14)	C1B—C6B—H6B	120.00
C1B—C2B—C8B	118.3 (14)	C5B—C6B—H6B	120.00
C3B—C2B—C8B	121.7 (14)	C1A—C7A—H7A	116.00
C2A—C3A—C4A	120.0 (5)	C8A—C7A—H7A	116.00
C2B—C3B—C4B	119.8 (15)	C9—C7B—H7B	120.00
C3A—C4A—C5A	120.0 (5)	C8B—C7B—H7B	120.00
C3B—C4B—C5B	120.2 (15)	C7A—C8A—H8A	118.00
C4A—C5A—C6A	120.0 (4)	C9—C8A—H8A	118.00
C4B—C5B—C6B	120.0 (16)	C2B—C8B—H8B	117.00
C1A—C6A—C5A	120.0 (4)	C7B—C8B—H8B	117.00
C1B—C6B—C5B	119.9 (15)	C9—C10—H10C	110.00
C1A—C7A—C8A	127.9 (4)	C9—C10—H10B	109.00
C8B—C7B—C9	119.9 (17)	C9—C10—H10A	109.00
C7A—C8A—C9	124.3 (5)	H10B—C10—H10C	109.00
C2B—C8B—C7B	126.1 (17)	H10A—C10—H10B	109.00
C7B—C9—C10	94.8 (9)	H10A—C10—H10C	110.00
O1—C9—C10	110.5 (2)	H11A—C11—H11B	110.00
O1—C9—C7B	127.9 (8)	O3—C11—H11A	109.00
C8A—C9—C10	117.9 (3)	O3—C11—H11B	109.00
P1—C9—O1	104.7 (2)	O3—C11—H11C	109.00
P1—C9—C8A	110.6 (3)	H11A—C11—H11C	109.00
P1—C9—C10	110.1 (2)	H11B—C11—H11C	109.00
P1—C9—C7B	108.2 (7)	H12B—C12—H12C	109.00
O1—C9—C8A	102.1 (3)	O4—C12—H12A	109.00
C2B—C1B—H1B	120.00	O4—C12—H12B	109.00
C6B—C1B—H1B	120.00	O4—C12—H12C	109.00
C1A—C2A—H2A	120.00	H12A—C12—H12B	110.00
C3A—C2A—H2A	120.00	H12A—C12—H12C	109.00
			
O2—P1—O3—C11	50.8 (3)	C6A—C1A—C2A—C3A	0.0 (8)
O4—P1—O3—C11	−73.1 (2)	C7A—C1A—C2A—C3A	−179.3 (5)
C9—P1—O3—C11	175.7 (2)	C2A—C1A—C6A—C5A	0.1 (7)
O2—P1—O4—C12	28.4 (3)	C7A—C1A—C6A—C5A	179.3 (4)
O3—P1—O4—C12	153.1 (3)	C2A—C1A—C7A—C8A	8.7 (8)
C9—P1—O4—C12	−98.0 (3)	C6A—C1A—C7A—C8A	−170.6 (5)
O2—P1—C9—O1	−60.4 (2)	C1A—C2A—C3A—C4A	−0.1 (9)
O2—P1—C9—C8A	−169.6 (3)	C2A—C3A—C4A—C5A	0.1 (10)
O2—P1—C9—C10	58.4 (3)	C3A—C4A—C5A—C6A	−0.1 (9)
O3—P1—C9—O1	174.29 (18)	C4A—C5A—C6A—C1A	0.0 (8)
O3—P1—C9—C8A	65.1 (3)	C1A—C7A—C8A—C9	179.0 (4)
O3—P1—C9—C10	−67.0 (2)	C7A—C8A—C9—P1	−93.6 (6)
O4—P1—C9—O1	65.8 (2)	C7A—C8A—C9—O1	155.5 (5)
O4—P1—C9—C8A	−43.4 (3)	C7A—C8A—C9—C10	34.2 (7)
O4—P1—C9—C10	−175.4 (2)		

### Hydrogen-bond geometry (Å, °)

**Table:**

*D*—H···*A*	*D*—H	H···*A*	*D*···*A*	*D*—H···*A*
O1—H1···O2^i^	0.82	1.90	2.721 (3)	176
C6B—H6B···Cg1^ii^	0.93	2.83	3.568 (17)	137
C6B—H6B···Cg2^ii^	0.93	2.94	3.652 (17)	134

Symmetry codes: (i) −*x*+1, −*y*+1, −*z*; (ii) −*x*+1/2, *y*+1/2, −*z*+1/2.
